# Epithelial‐to‐mesenchymal transition signature assessment in colorectal cancer quantifies tumour stromal content rather than true transition

**DOI:** 10.1002/path.5155

**Published:** 2018-11-16

**Authors:** Amy MB McCorry, Maurice B Loughrey, Daniel B Longley, Mark Lawler, Philip D Dunne

**Affiliations:** ^1^ Centre for Cancer Research and Cell Biology Queen's University Belfast UK; ^2^ Cellular Pathology Belfast Health and Social Care Trust Belfast UK

**Keywords:** bioinformatics, gene signatures, colorectal cancer, EMT, fibroblasts, pathology, stroma

## Abstract

The process of epithelial‐to‐mesenchymal transition (EMT) in cancer is a well‐described process whereby epithelial tumour cells undergo molecular/phenotypic changes and transition to a mesenchymal biology. To aid in the transcriptional characterisation of this process, gene expression signatures have been developed that attribute a relative EMT score to samples in a given cohort. We demonstrate how such EMT signatures can identify epithelial cell line models with high levels of transition but also highlight that, unsurprisingly, fibroblast cell lines, which are inherently mesenchymal, have a higher EMT score relative to any epithelial cell line studied. In line with these data, we demonstrate how increased tumour stromal composition, and reduced epithelial cellularity, significantly correlates with increasing EMT signature score, which is evident using either *in silico* subtyping analysis (*p* < 0.00001) or *in situ* histopathological characterisation (*p* < 0.001). Considered together, these results reinforce the importance not only of interdisciplinary research to correctly define the nature of EMT biology but also the requirement for a cadre of multidisciplinary researchers who can analyse and interpret the underlying pathological, bioinformatic and molecular data that are essential for advancing our understanding of the malignant process. © 2018 The Authors. *The Journal of Pathology* published by John Wiley & Sons Ltd on behalf of Pathological Society of Great Britain and Ireland.

## Introduction

Due to the decreasing cost of generating molecular information, large repositories [such as The Cancer Genome Atlas (TCGA) and Gene Expression Omnibus (GEO)] now provide access to semi‐resolved molecular data. This explosion in data availability has created a bottleneck in the cancer research pipeline as a large proportion of traditional ‘wet‐lab’ molecular biologists are unable to independently perform the complex bioinformatic interrogation required to maximise the value of this freely available data. Although pathologists perform diagnostic assessments of tissue following initial processing, the biological interrogation of these samples is frequently undertaken in a manner that fails to account for the fundamental tumour pathology by researchers with a predominantly computational biology or bioinformatics background, with input from molecular biologists. The multidimensional nature of the sample‐processing pipeline, involving input from pathologists, geneticists and bioinformaticians, coupled with the data capture, analysis and elucidation can result in different biological interpretations that may be irrelevant to the disease setting because they are focussed on the incorrect cell‐of‐origin. To overcome this, collaboration and communication between all the sub‐disciplines involved in data generation and analysis is required.

Through examples, this study examines the use of transcriptional classification, with a focus on epithelial‐to‐mesenchymal transition (EMT) gene expression signatures in colorectal cancer (CRC) as Morris and Kopetz recently commented on how the ‘distinctions between mesenchymal and EMT signatures are commonly blurred in the gene expression literature’ 1. In this study, we demonstrate how a disconnect between pathological/experimental ‘wet‐lab’ research and data processing/bioinformatics ‘dry‐lab’ analysis can introduce potential confounding errors in the interpretation of gene expression signatures in tumour cohorts and the understanding of molecular subtypes in cancer.

## Materials and methods

### CRC transcriptional profiles

All public datasets were downloaded from GEO (http://www.ncbi.nlm.nih.gov/geo/) or ArrayExpress (http://www.ebi.ac.uk/arrayexpress/) using the indicated accession numbers:

*E‐MTAB‐863*: 215 stage II colon tumours transcriptionally profiled using the Almac CRC Disease Specific Array (DSA; Almac, Craigavon, Northern Ireland, UK); *GSE103479*: 156 stage II/III colorectal tumours transcriptionally profiled using the Almac Xcel array (Almac, Craigavon, Northern Ireland, UK); and *GSE39396*: Fluorescence‐Activated Cell Sorting (FACS) separated six fresh colorectal tumours into endothelial (CD31^+^), epithelial (EPCAM^+^), leukocyte (CD45^+^) and fibroblast (FAP^+^) populations, followed by transcriptional profiling using the Affymetrix HT HG‐U133 Plus PM array (Santa Clara, CA, USA).

### Cancer Cell Line Encyclopedia

RMA‐normalised mRNA expression microarray data were obtained from the Cancer Cell Line Encyclopedia (CCLE) via https://data.broadinstitute.org/ccle_legacy_data/mRNA_expression/CCLE_Expression_2012-09-29.res.

### ssGSEA EMT signature score

Single‐sample gene set enrichment analysis was performed using the ssGSEAProjection module on GenePattern (https://genepattern.broadinstitute.org/) with the EMT gene set (HALLMARK_EPITHELIAL_MESENCHYMAL_TRANSITION; MSigDB v6.1).

More details of the datasets and methods for data normalisation; clustering analysis; and calculation of relative gene expression levels, signature scores for cancer‐associated fibroblast (CAF) and endothelial cells, immune and stromal cell correlations, consensus molecular subtype (CMS) and epithelial cell fraction are provided in supplementary material, Supplementary materials and methods.

## Results

### Assessment of EMT signature score in cell line models

**Figure 1:**
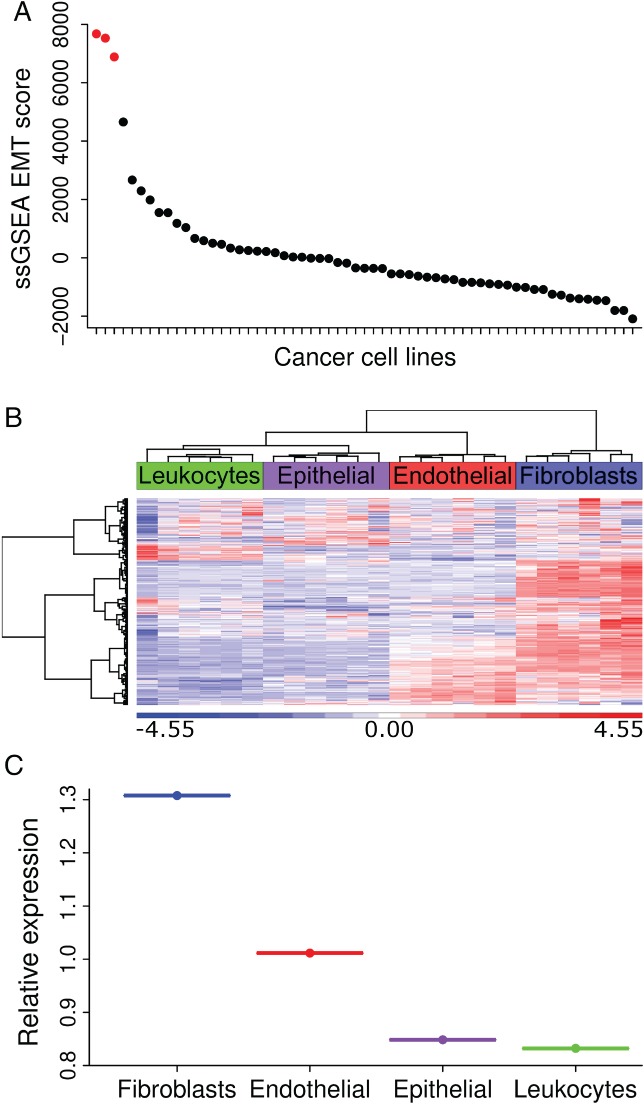
(A) Microarray profiles from the 61 CRC cell line models within the CCLE were scored using the ssGSEA EMT signature, followed by ranking from high to low according to the resulting EMT score. Red dots indicate cell lines with fibroblast or connective tissue origin. (B) Semi‐supervised hierarchical clustering of the EMT signature genes in the GSE39396 cohort demonstrates the stromal (particularly fibroblast) origin of transcription in CRC. GSE39396 contains *n* = 24 transcriptional profiles from isolated epithelial, endothelial, leukocyte and fibroblast lineages from six freshly resected colorectal tumour tissue samples. (C) Median relative expression of EMT signature genes in each individual lineage as in (B).

The CCLE provides molecular data from ∼1500 cancer cell lines. Using this database, we selected transcriptional profiles for 61 CRC cell line models for the assessment of EMT signature scores using the ssGSEA EMT Hallmark signature 2, which was chosen because it is derived from multiple founder datasets, followed by testing and independent validation using experimental data. Although there is no defined threshold for what constitutes a ‘high’ EMT score, the majority of cell lines had an ssGSEA EMT score below the arbitrary figure of 1000, while three cell lines demonstrated EMT scores >6000 (Hs 698.T: 6884, Hs 255.T: 7676, Hs 675.T: 6884; Table 1 and Figure 1A). Investigation of the origin/histology of these cell lines demonstrated that, unlike the other epithelial cell lines investigated, these three were described as either fibroblasts or connective tissue from the American Tissue Culture Collection (ATCC). These cell line findings highlight an important issue. Although fibroblast (non‐epithelial) cell populations can easily be excluded from a cell line experiment aimed at identifying EMT signatures in epithelial cells, this is not possible when dealing with tumour samples. Even with the attempted enrichment for the epithelial cell population by macrodissection, there is inevitable and innate variation in fibroblast abundance within the tumour microenvironment (TME). Not considering this variation may result in an inaccurate interpretation of EMT data derived from samples within a tumour tissue cohort.

**Table 1:** Histological characteristics and lineage information for a subset of the CCLE cell lines according to ssGSEA EMT signature score

Cell line	EMT score	Description
Hs 698.T	7675.53	Colon; derived from metastatic site: connective tissue
Hs 255.T	7527.33	Colon; morphology: fibroblast
Hs 675.T	6884.19	Colon; morphology: fibroblast
MDST8	4654.13	Colon; cell type: epithelial‐like
SNU‐1197	2665.23	Colon; cellular morphology: epithelial
GP2d	−1452.18	Colon; cell type: epithelial
SNU‐283	−1467	Colon; cellular morphology: epithelial
SW403	−1804.08	Colon; morphology: epithelial
SNU‐283	−1804.47	Colon; cellular morphology: lymphoblast‐like, round
CCK‐81	−2091.15	Colon; adenocarcinoma

The upper section displays EMT scores and information for the five highest ranking cell lines, and the lower section displays EMT scores and information for the five lowest ranking cell lines.

To validate our hypothesis, we assessed the EMT signature genes in a 24‐sample discovery cohort, derived from six CRC patients, where fibroblast, endothelial cell, epithelial cell and leukocyte populations were isolated and transcriptionally profiled. This assessment demonstrated that >75% of the genes within the EMT signature were associated with non‐epithelial lineages of the TME (Figure 1B), predominantly the CAF component (Figure 1C).

### EMT signature score is associated with stromal components of the TME

**Figure 2:**
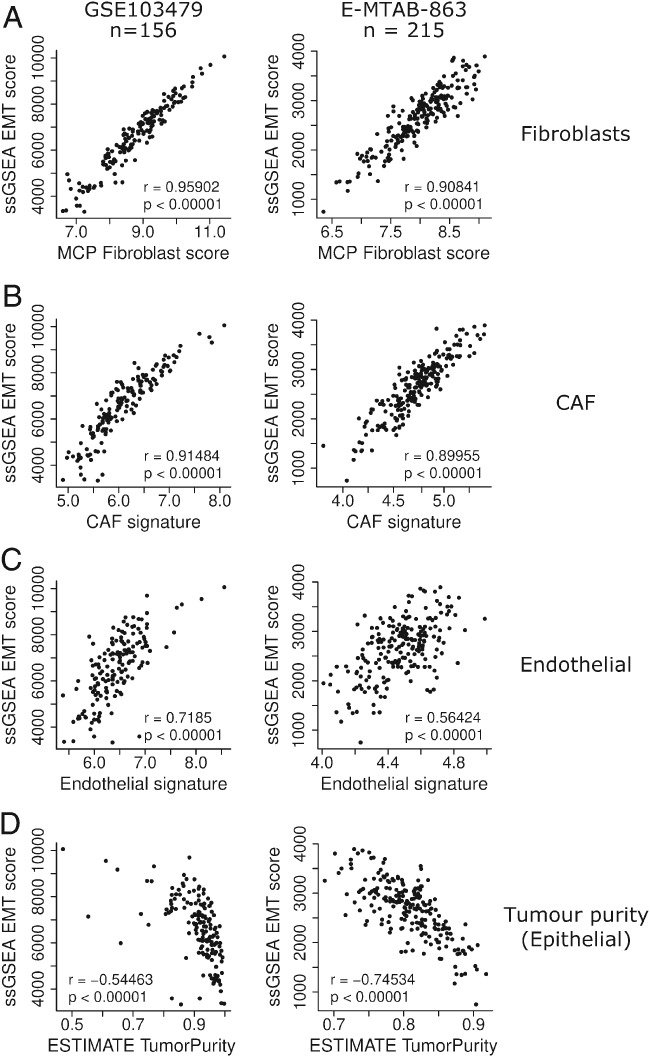
EMT signatures classification of microarray profiles from two independent CRC cohorts (E‐MTAB‐863 and GSE103479) correlated to: (A) MCP‐counter fibroblast score, (B) CAF signature score, (C) endothelial signature score and (D) tumour purity (epithelial) ESTIMATE score. E‐MTAB‐863 contains microarray profiles from 215 stage II FFPE colon cancer tumours, transcriptionally profiled using the Almac Colorectal Cancer DSA platform. GSE103479 contains microarray profiles from 156 stage II and III fresh frozen colon cancer tumours, transcriptionally profiled using the Almac Xcel array platform.

Based on these preliminary data, we attempted to comprehensively validate our findings in two cohorts: GSE103479 (*n* = 156) and E‐MTAB‐863 (*n* = 215). We performed ssGSEA EMT signature assessment, followed by correlation with fibroblast‐specific scores from the microenvironment cell populations counter (MCP) 3 alongside the CAF and cancer‐associated endothelial (CAE) signatures previously generated from CRC tissue that underwent FACS enrichment and subsequent transcriptional profiling 4, 5. These analyses confirmed that the EMT signature is associated with the stromal component of a tumour, with a significant correlation between EMT scores and MCP‐fibroblast (*p* < 0.00001; Figure 2A), CAF (*p* < 0.00001; Figure 2B) and CAE levels (*p* < 0.00001; Figure 2C). Importantly, the EMT score was negatively correlated with epithelial cell levels (assessed by the tumour purity ESTIMATE algorithm) 6 (*p* < 0.00001; Figure 2D, see supplementary material, Figures S1 and S2), further supporting the hypothesis that tumoural EMT assessment is a measure of stromal abundance rather than epithelial transition.

### Pathological assessment of tumour tissue according to EMT signature score

**Figure 3:**
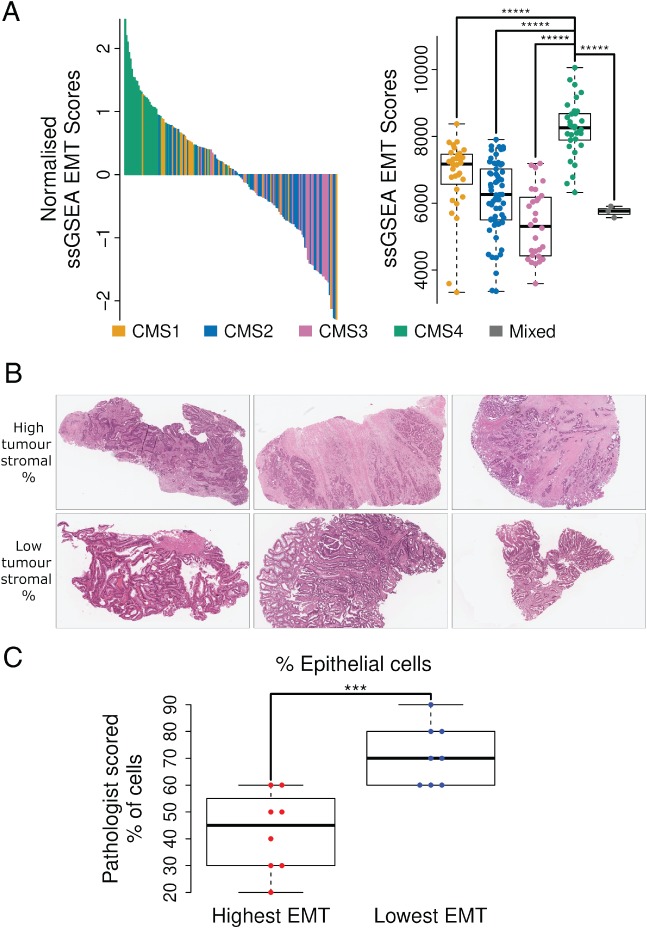
(A) CMS classification of the GSE103479 cohort and alignment with normalised ssGSEA EMT signature score (left). Boxplot detailing ssGSEA raw scores for individual samples according to CMS classification, indicating median, upper/lower quartile and max/min values. *****p < 0.00001. (B) H&E images of tumour samples with the highest and lowest ssGSEA EMT signature scores. (C) Pathologist scoring, blinded to tumour EMT classification, indicates significant association between EMT signature score and tumour/stromal content of tumour tissue. ***p < 0.001.

Transcriptomic interrogation of CRCs has demonstrated four CMSs 7. Two of these correspond to well‐characterised histological subtypes: CMS1 (immune‐rich) and CMS4 (stromal, particularly fibroblast‐rich), alongside two epithelial‐rich subtypes: CMS2 and CMS3. Using the GSE103479 cohort, in line with findings from the CMS study, we confirmed the significant overlap between high EMT score and the fibroblast‐rich CMS4 subtype (Figure 3A. *p* < 0.00001) and low EMT scores associated with the epithelial‐rich CMS2/CMS3 tumour subtypes. Next, we performed an assessment of the CRC intrinsic subtypes (CRIS) that previously identified increased intrinsic EMT signalling within CRIS‐B tumours 8, 9. Results indicated that, while CMS4 tumours were distributed across all CRIS subtypes, CRIS‐D contained the largest relative number of CMS4s (44%; see supplementary material, Figure S3A). CRIS‐B tumours had a significantly higher overall EMT score compared to CRIS‐A (*p* < 0.01), CRIS‐C (*p* < 0.05) and CRIS‐E (*p* < 0.01) (see supplementary material, Figure S3B), likely due to their high intrinsic EMT signalling. The highest EMT‐scoring tumours were significantly associated with the stromal‐rich CMS4 subtype (*p* < 0.00001 compared to all other CMS subtypes), with only one CMS4 tumour scoring below the median, reinforcing the hypothesis that the stroma makes a more significant contribution to an individual tumour's EMT score regardless of intrinsic subtype (Figure 3A, see supplementary material, Figure S3B).

We then selected cases with the highest (*n* = 8) and lowest (*n* = 8) ssGSEA EMT signature scores from this cohort and performed a pathological assessment of the epithelial and stromal composition of the tumour tissue used to generate these transcriptional profiles, with the assessing pathologist blinded to all transcriptional data and classifications (Figure 3B). In support of our hypothesis, tumours with the lowest EMT scores were significantly associated with the highest epithelial cellularity (Figure 3C. *p* < 0.001; tumour epithelial cellularity 60–90%), even when samples containing high immune infiltrate (making cellularity estimation difficult) are considered (see supplementary material, Figure S5. *p* < 0.01).

## Discussion

The EMT‐like molecular subgroup has been identified in a number of cancers, including those arising in the colorectum, breast, ovary, prostate and lung. 10 This subgroup is associated with poor prognosis and has been widely misinterpreted to represent a class of tumours where epithelial cells have undergone a widespread transition to a mesenchymal phenotype, resulting in more invasive and aggressive tumour behaviour. The use of inaccurate nomenclature to describe these signatures has been further confounded by the increasing distance between the ‘end user’ interpreting the findings and the preceding steps of tissue procurement and handling, molecular profiling and data generation/analysis. Although all these steps may still be performed by the wet‐lab biologist when low‐throughput single‐biomarker studies are required, given the multidisciplinary approach required for comprehensive omics‐level tumour profiling analysis, these steps are increasingly being performed by core units, service providers or stand‐alone bioinformatics departments/units. This potential lack of multidisciplinary oversight, from tissue collection to bioinformatics reporting, can result in the misinterpretation of the underlying pathology/biology ensuing from the molecular data.

The data presented here do not challenge the clinical importance of the EMT subtype 10, 11; rather, they highlight how the alignment of predefined signatures, such as EMT, in either cancer cell lines or cancer tissue samples can result in the identification of similar subgroups (EMT‐high) based on two inherently different underlying biologies, namely, true EMT or innate high cancer fibroblast composition. Our data are in line with previous findings from the CMS consortium, which demonstrated an enrichment of CAF/CAE in the stromal‐rich CMS4 subtype (or equivocal Stem/Serrated/Mesenchymal subtypes) using either *in silico* or histological assessment 4, 7. Importantly, however, we now demonstrate that the correlation between stromal infiltration and EMT signature is predominantly due to the fibroblast content *per se* and not a transition of epithelial cells within the tumour. We do not definitively rule out that there could be some contribution to the EMT score from epithelial cells undergoing true EMT within a tumour mass, but we consider this a relatively rare event. We observed that even the highest EMT scores achieved by epithelial cells were low compared to those seen in fibroblasts, suggesting that, in line with our previous work on the stability of subtypes at multiple regions of tumour tissue, 12 subtle changes in tumour–stroma ratio can contribute more significantly to the EMT signature than epithelial cells undergoing transition. Thus, macrodissection of tumour samples to enrich for the epithelial tumour cell population is likely to negatively influence the EMT score. Further detailed investigation of the stromal‐rich CMS4 subtype in CRC has demonstrated the clinical importance of not only the CAF component but also the interplay between the milieu of inflammatory and endothelial lineages and the associated cytokine production 13. Our study, and that of Becht and colleagues 13, emphasises the importance of integrating comprehensive pathological assessment with molecular subtyping in order to fully exploit and most accurately interpret such data and their clinical implications.

## Author contributions statement

AMBM performed analyses, generated figures and interpreted data. MBL conducted a pathological assessment blinded to transcriptional classification of each sample and interpreted data. DBL provided CRC tissue samples, molecular profiles and interpretation of the data. ML interpreted data, supervised the study and revised the manuscript. PDD designed and supervised the study, performed analysis, conducted pathological assessment, interpreted data and drafted the manuscript. All authors contributed to the final manuscript.

**SUPPLEMENTARY MATERIAL ONLINE**

**Supplementary materials and methods**

**Figure S1.** Comprehensive in silico histology assessment of the GSE103479 cohort using MCP‐counter

**Figure S2.** Comprehensive in silico histology assessment of the E‐MTAB‐863 cohort using MCP‐counter

**Figure S3.** CRIS profiling of the GSE103479 cohort

**Figure S4.** CMS and CRIS classifications using thresholds from original studies

**Figure S5.** Pathologist scoring, blinded to tumour EMT classification, indicates significant association between EMT signature score and tumour/stromal content of tumour tissue

## Supporting information


**Supplementary materials and methods**

**Figure S1.** Comprehensive in silico histology assessment of the GSE103479 cohort using MCP‐counter
**Figure S2.** Comprehensive in silico histology assessment of the E‐MTAB‐863 cohort using MCP‐counter
**Figure S3.** CRIS profiling of the GSE103479 cohort
**Figure S4.** CMS and CRIS classifications using thresholds from original studies
**Figure S5.** Pathologist scoring, blinded to tumour EMT classification, indicates significant association between EMT signature score and tumour/stromal content of tumour tissue. **p < 0.01
