# Supplementary material for: Epithelial‐to‐mesenchymal transition signature assessment in colorectal cancer quantifies tumour stromal content rather than true transition
Source: J Pathol. 2018 Nov 16;246(4):422–6. doi: 10.1002/path.5155 (PMC6282832; doi:10.1002/path.5155)
Supplement: Supplementary file 1 — Supplementary materials and methods [file PATH-246-422-s001.docx]

**Epithelial-to-mesenchymal transition signature assessment in colorectal**

**cancer quantifies tumour stromal content rather than true transition**

**McCorry AMB *et al.* J Pathol 2018 (DOI: 10.1002/path.5155)**

**Supplementary materials and methods**

Reference numbers refer to the main text list

**Patient Cohort:** GSE103479 was assembled from an initial cohort of stage II and III disease from four European Centres (Vall d’Hebron Institute of Oncology, Barcelona, Spain; St Vincent’s University Hospital, Dublin, Ireland; University of Florence, Florence, Italy; and University of Aberdeen, Aberdeen, United Kingdom). This work was approved by the Medicine, Dentistry, and Biomedical Sciences School Ethics Committee of Queen’s University Belfast (ref: 12/12v4).

**Data availability and normalisation:** Raw data of GSE103479 and E-MTAB-863 were obtained from GEO (https://www.ncbi.nlm.nih.gov/geo/) and ArrayExpress (https://www.ebi.ac.uk/arrayexpress/). CEL files were imported into Partek Genomics Suite (PGS v6.6; St. Louis, MO, USA) and were normalised using Robust Multi-array Average (RMA). Both cohorts were examined using principal component analysis (PCA) and a batch effect by scan date was identified in the GSE103479 cohort and was removed using the “Remove Batch Effect” tool in PGS. For GSE39396 and the cell line microarray data obtained from the CCLE the processed data, which had been RMA normalised, was used.

**Clustering analysis:** Partek Genomics Suite (v6.6) was used to standardise the data by generating row-centred Z scores and perform hierarchical clustering using Ward’s linkage and Euclidean distance.

**Relative expression of EMT genes:** Relative expression was calculated following the same method used by Dunne *et al.*[8] In brief, the cell-specific average expression for each probeset, associated with one of the 200 genes from the EMT gene set, was divided by the overall average for that probeset then median value for each cell type was calculated.

**CAF, Endothelial, ESTIMATE and MCP scores:** Prior to analysis each dataset was collapsed to the probeset with the highest mean value for each gene using the “collapseRows” function from the WGCNA R package (v1.61)[14]. CAF and Endothelial signatures scores were calculated as mean expression of the genes within each signature from Isella *et al.*[9] for each sample. MCP and ESTIMATE scores were generated using the R packages MCPcounter (v1.1.0)[3] and estimate (v1.0.13)[6].

**Immune and stromal cell correlations:** For all correlations the r and p values shown are calculated using the “cor.test” function from the stats R package using the “pearson” method. The P values generated are based on the *t*-statistic for each *r* value.

**CMS and CRIS classifications:** Consensus Molecular Subtype (CMS) classification employed the “classifyCMS.RF” function with default settings from the CMSclassifier R package [7] obtained from GitHub (https://github.com/Sage-Bionetworks/CMSclassifier/) in R (v3.3.2) (https://www.R-project.org). CRC intrinsic subtype (CRIS) classifications [9] were created using the NearestTemplatePrediction module (v4) on GenePattern (https://genepattern.broadinstitute.org/) [15]. The CRIS genes used contained an addition 26 updated gene symbols obtained from Supplementary Data 7 from Dunne *et al.*[8] For both subtypes the nearest predicted class was used. Data from classifications using the original thresholds for each method, therefore containing unclassified samples, are provided in Supplementary Figure S4.

**Epithelial cell fraction:** The P value for the significance comparison of the fraction of epithelial cells between the samples with the highest and lowest EMT scores was derived from Student’s *t*-test (calculated using the “t.test” function within the R stats package).
