# Supplementary material for: Epithelial‐to‐mesenchymal transition signature assessment in colorectal cancer quantifies tumour stromal content rather than true transition
Source: J Pathol. 2018 Nov 16;246(4):422–6. doi: 10.1002/path.5155 (PMC6282832; doi:10.1002/path.5155)
Supplement: Supplementary file 2 — Figure S1. Comprehensive in silico histology assessment of the GSE103479 cohort using MCP‐counter Figure S2. Comprehensive in silico histology assessment of the E‐MTAB‐863 cohort using MCP‐counter Figure S3. CRIS profiling of the GSE103479 cohort Figure S4. CMS and CRIS classifications using thresholds from original studies Figure S5. Pathologist scoring, blinded to tumour EMT classification, indicates significant association between EMT signature score and tumour/stromal content of tumour tissue. **p < 0.01 [file PATH-246-422-s002.pdf]

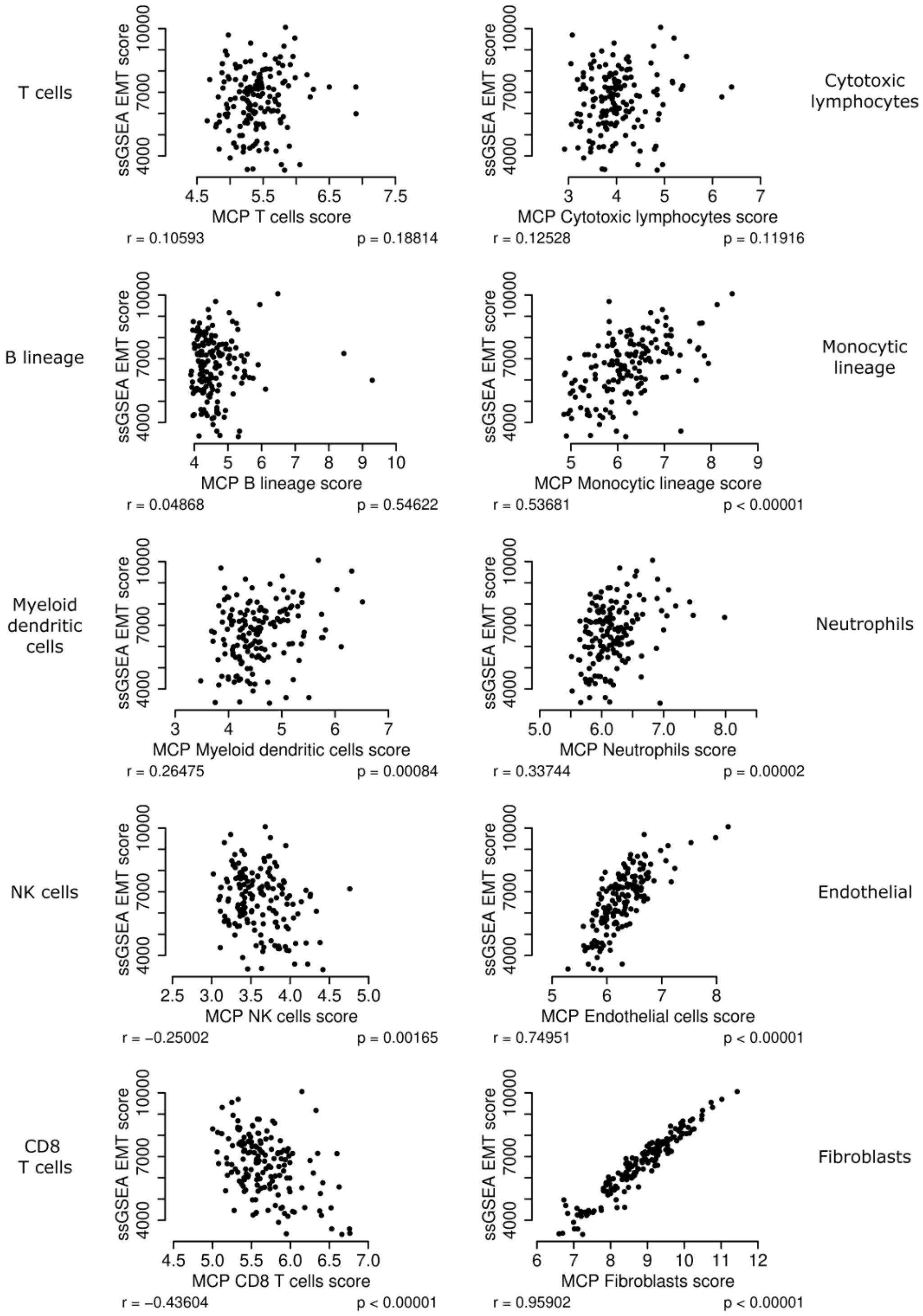

Supplementary Figure S1. Comprehensive *in silico* histology assessment of the GSE103479 cohort using MCP-counter.

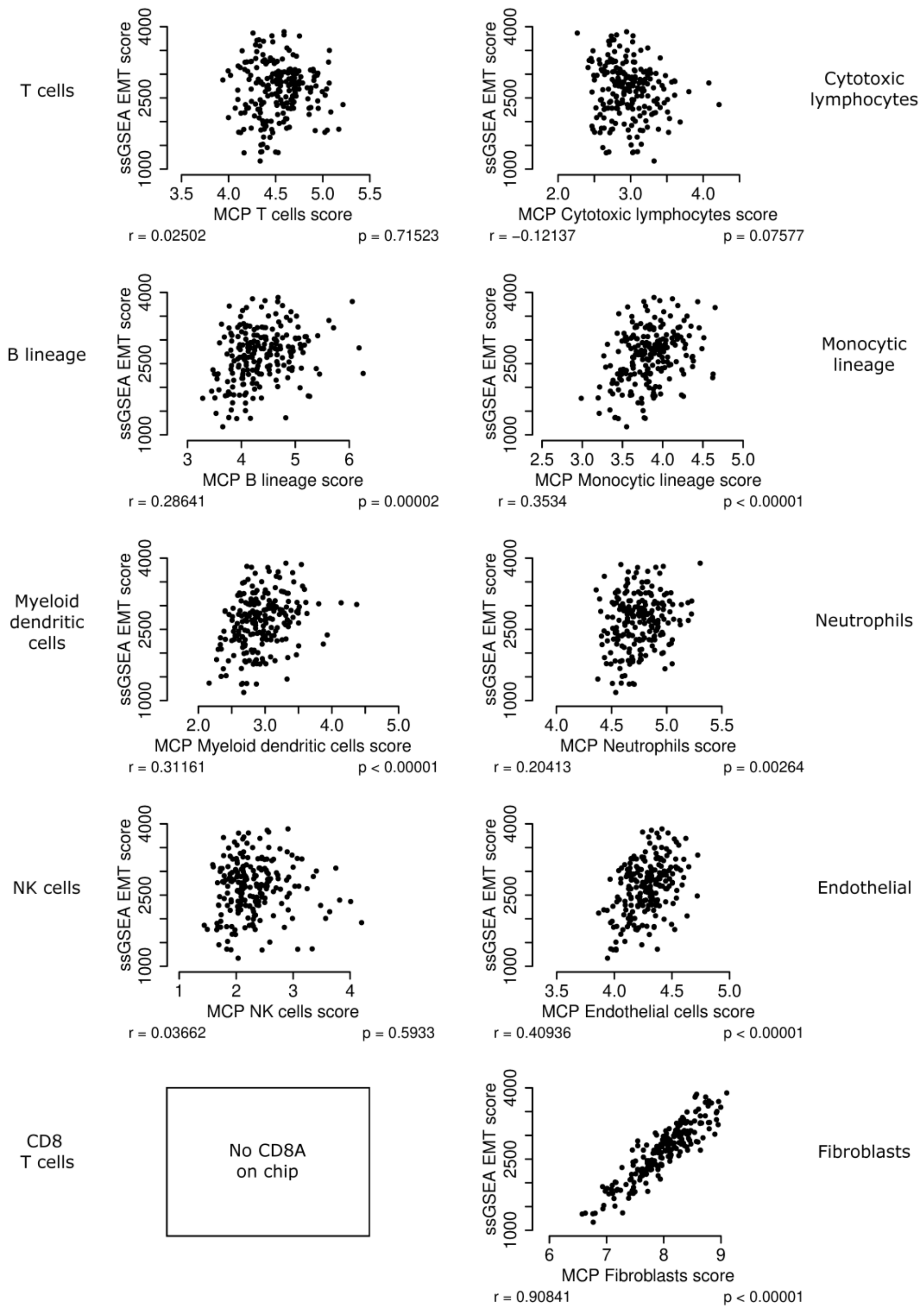

Supplementary Figure S2. Comprehensive *in silico* histology assessment of the E-MTAB-863 cohort using MCP-counter.

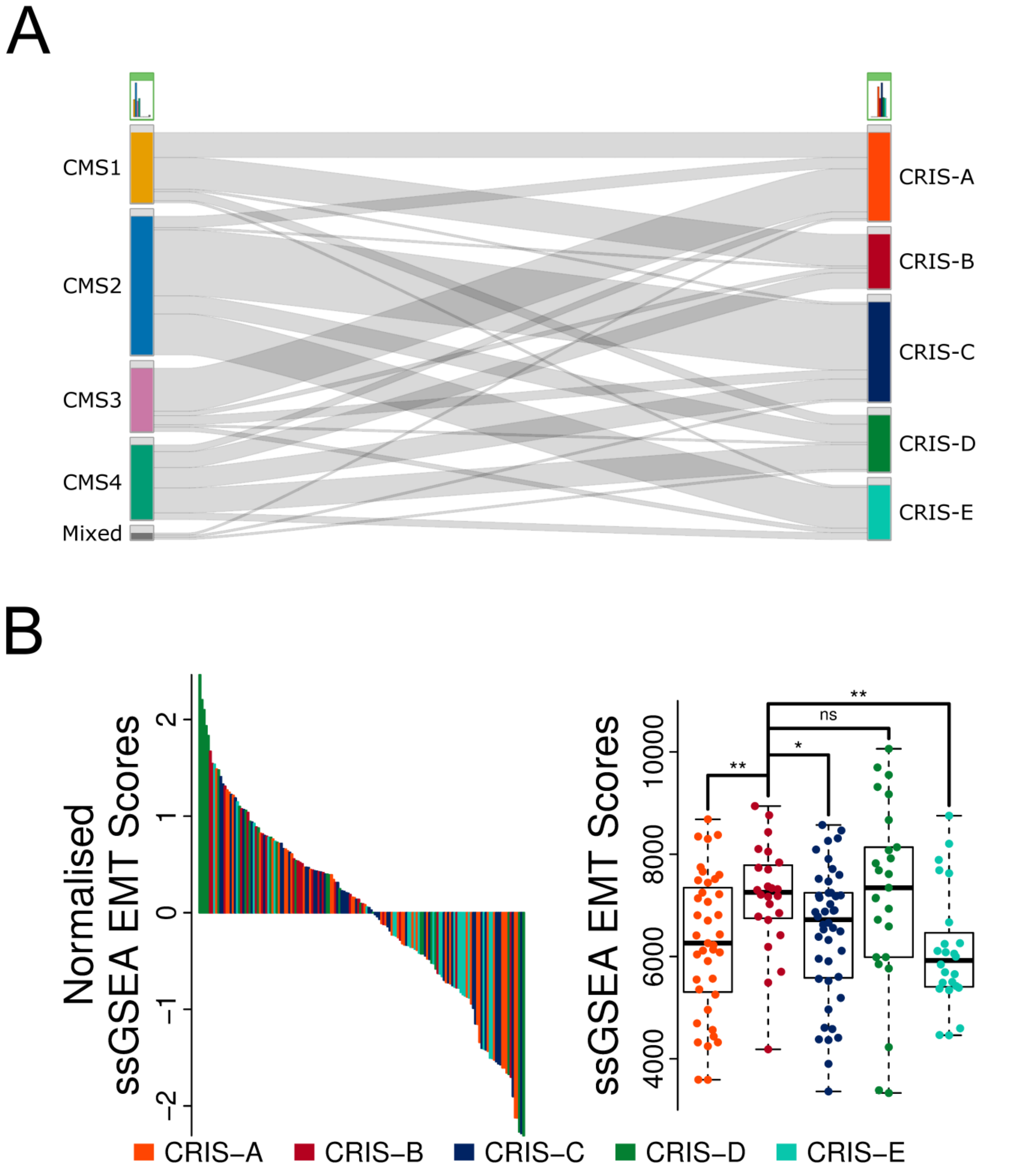

Supplementary Figure S3. CRIS profiling of the GSE103479 cohort. **A.** Distribution of CMS and CRIS for each individual sample. **B.** CRIS classification of the GSE103479 cohort and alignment with normalised ssGSEA EMT signature score. Boxplot detailing ssGSEA raw scores for individual samples according to CRIS classification, indicating median, upper/lower quartile and max/min values. * = $P < 0.05$, ** = $P < 0.01$, ns = not significant.

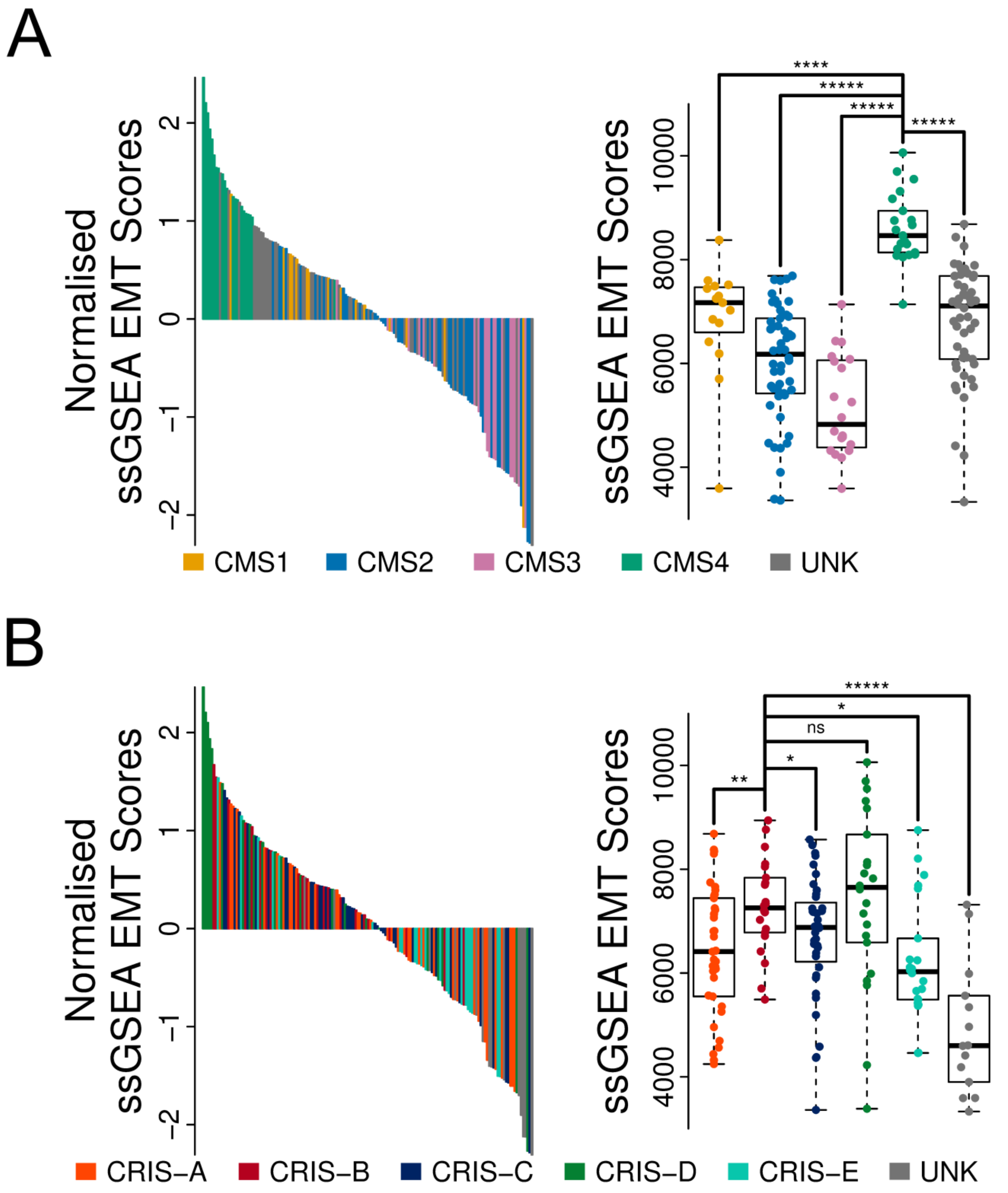

Supplementary Figure S4. CMS and CRIS classifications using thresholds from original studies **A.** CMS classification of the GSE103479 cohort, with unclassified samples designated as UNK, and alignment with normalised ssGSEA EMT signature score (Left). Boxplot detailing ssGSEA raw scores for individual samples according to CMS classification, indicating median, upper/lower quartile and max/min values. **** = $P < 0.0001$, ***** = $P < 0.00001$. **B.** CRIS classification of the GSE103479 cohort, with unclassified samples designated as UNK, and alignment with normalised ssGSEA EMT signature score (Left). Boxplot detailing ssGSEA raw scores for individual samples according to CMS classification, indicating median, upper/lower quartile and max/min values. * = $P < 0.05$, ** = $P < 0.01$, ***** = $P < 0.00001$, ns = not significant.

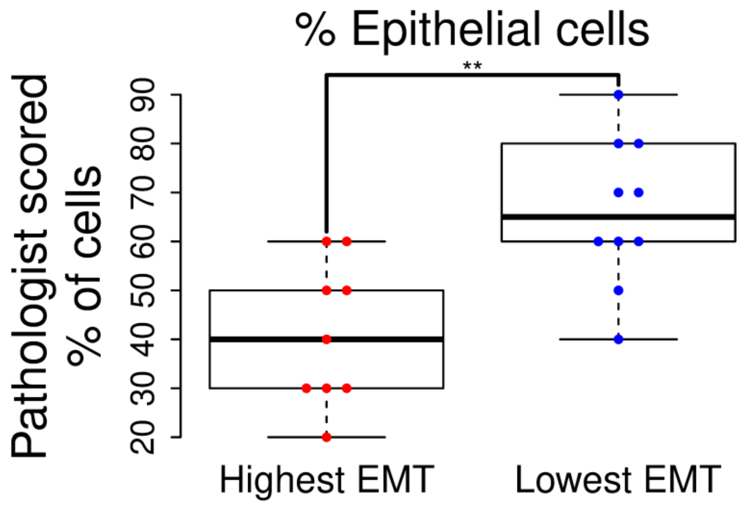

Supplementary Figure S5. Pathologist scoring, blinded to tumour EMT classification, indicates significant association between EMT signature score and tumour/stromal content of tumour tissue. ** = $P < 0.01$.
